# Building and analyzing protein interactome networks by cross-species comparisons

**DOI:** 10.1186/1752-0509-4-36

**Published:** 2010-03-30

**Authors:** Amy M Wiles, Mark Doderer, Jianhua Ruan, Ting-Ting Gu, Dashnamoorthy Ravi, Barron Blackman, Alexander JR Bishop

**Affiliations:** 1 Greehey Children's Cancer Research Institute, The University of Texas Health Science Center at San Antonio, San Antonio, TX 78229, USA; 2 Department of Cellular and Structural Biology, The University of Texas Health Science Center at San Antonio, San Antonio, TX 78229, USA; 3 Department of Computer Science, The University of Texas at San Antonio, San Antonio, TX 78249, USA

## Abstract

**Background:**

A genomic catalogue of protein-protein interactions is a rich source of information, particularly for exploring the relationships between proteins. Numerous systems-wide and small-scale experiments have been conducted to identify interactions; however, our knowledge of all interactions for any one species is incomplete, and alternative means to expand these network maps is needed. We therefore took a comparative biology approach to predict protein-protein interactions across five species (human, mouse, fly, worm, and yeast) and developed InterologFinder for research biologists to easily navigate this data. We also developed a confidence score for interactions based on available experimental evidence and conservation across species.

**Results:**

The connectivity of the resultant networks was determined to have scale-free distribution, small-world properties, and increased local modularity, indicating that the added interactions do not disrupt our current understanding of protein network structures. We show examples of how these improved interactomes can be used to analyze a genome-scale dataset (RNAi screen) and to assign new function to proteins. Predicted interactions within this dataset were tested by co-immunoprecipitation, resulting in a high rate of validation, suggesting the high quality of networks produced.

**Conclusions:**

Protein-protein interactions were predicted in five species, based on orthology. An InteroScore, a score accounting for homology, number of orthologues with evidence of interactions, and number of unique observations of interactions, is given to each known and predicted interaction. Our website http://www.interologfinder.org provides research biologists intuitive access to this data.

## Background

Proteins often physically interact to carry out their functions within living cells. A protein-protein interactome, or a protein-protein interaction (PPI) network, is the collection of these interactions between proteins in a single organism. The utility of generating a high quality PPI network with significant protein coverage for any species is manifold. It has been suggested that if two proteins interact, then they are likely to have related functions [1]. Therefore, perhaps the greatest benefit of a comprehensive PPI map will be to provide insight into the biology of proteins with no known function, a significant issue for all species in this "post-genomic era."

Systematic identification of PPI has provided new insights into the relationships between both individual proteins and the processes within which they are involved. There have been several studies aimed at determining the full set of PPI for multiple model organisms [2-8], as well as for humans [9-11], and a wide range of publications on PPI at a much smaller scale. The strength of an interactome is in the quality and extent of the available data. Although there is a considerable amount of interaction data available for organisms such as yeast, worm, fly, human, and to a lesser extent mouse, unfortunately there is an underrepresentation of the potential PPI, with a majority of proteins not included in the current maps for any one species. The exception is yeast, which is estimated to have 18,000 ± 4500 interactions [12] encompassing 84% of the known proteome. In the absence of a technological advance that would improve the coverage of PPI identification for a single species, alternative approaches need to be incorporated to speed the development of more complete PPI network maps.

One way to address the current deficiencies within available PPI data is to examine orthologues of interacting proteins in other organisms and predict possible interologues (conserved PPI between sets of conserved orthologues). The basis of this comparison is that proteins encoded by orthologous genes maintaining a conserved function also maintain most, if not all, PPI with other conserved proteins. In order to predict interologues by conservation, one must first obtain an accurate set of orthologues. In general, the BLAST sequence alignment algorithm [13] is commonly used for identifying interspecies orthologues due to being computationally inexpensive, albeit less robust than algorithms such as Smith-Waterman [14]. With this cross-species mapping, conserved interologues can be identified and predicted as has been reported by others [15-21]. The interaction prediction database STRING [19,22-24] uses Clusters of Orthologous Groups of proteins (COGs) for orthologues between species.

Alternatively, Michaut *et al*. [22] predicted interactions in a multitude of organisms using pair-wise Smith-Waterman similarity to determine orthology, with a best reciprocal hit approach, resulting in all "orthologues" existing in a one-to-one relationship. A more powerful approach for orthology mapping was used by Ensembl [25], where a combination of BLAST and Smith-Waterman was used for alignments, followed by reconciliation using the phylogenetic tree of all species in their database. This allows for more than one-to-one orthologues, a feature of which we take advantage to generate comprehensive, predicted PPI network maps.

Considering the recent use of protein interactomes in defining pathways or predicting gene involvement in disease on the premise of "guilt-by-association," expanding our knowledge of PPIs would be of benefit to health sciences [26,27]. Because the number of interactions observed within a single species is very limited, we have endeavored to predict interologues from known interactions in other species. Through intra-species and inter-species analysis, we predict new PPI for human, mouse, fly, worm, and yeast. We then assign a confidence measurement to each interaction based on the level of conservation of the interaction across multiple species and the number of supporting experiments. This measurement allows interologues comprised of one-to-many and many-to-many orthologues to be scored, although here, we score them lower based on their level of homology. In addition, we implement PPI predictions to interpret biological data. Because the software Cytoscape [28] offers highly flexible network visualization, we provide all of our results in a Cytoscape compatible format as supplementary data. We also developed a web interface (interologfinder.org) for downloading pre-computed files and exploring user-specified genes of interest, for which both browsing and Cytoscape files are available.

## Results

### Comparison of Orthologues

**Figure 1:**
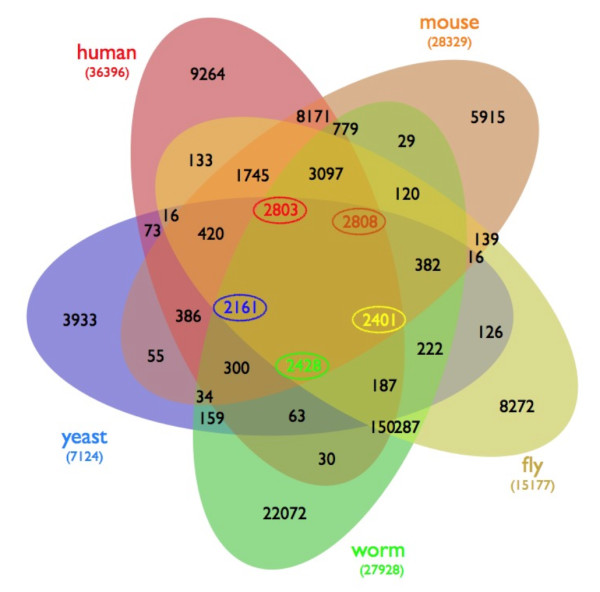
**Five-way Venn Diagram of orthologous genes in five species**. Orthologues in human (red), mouse (orange), fly (yellow), worm (green), and yeast (blue). Numbers of genes overlapping between all five species are given in their respective colors and encircled. All other groups of orthologous genes only show the number of genes found in the organism with the largest number in that group. Orthologues were retrieved from Ensembl 50.

In order to facilitate the comparison of interactions across species, we first surveyed the amount of gene conservation between species. Orthologue data from Ensembl 50 were compared between five species: human, mouse, fly, worm, and yeast, and the number of orthologues between species were determined for those genes whose status Ensembl annotated as "known." Figure 1 shows the overlap between the organisms as a five-way Venn diagram. Human and mouse are the most closely related organisms we are investigating and as such, demonstrate a high level of gene conservation. Yeast, however, shares less than half of its genome with the four animal species in this study. A large majority of genes annotated "novel" was found to be unique to their species. For instance 9,390 human genes were "novel" (as noted by Ensembl), and 9,165 of these did not have orthologues in any of the other four species, bringing their authenticity into question.

For those genes that are conserved in all five species, Gene Ontology (GO) analysis reveals an overrepresentation of a wide variety of metabolic, biosynthetic, and DNA repair processes (Additional file 1: Table S1). These same GO annotations are consequently under-represented in the non-conserved genes for each species. In contrast, processes such as signal transduction, extracellular communication, integral membrane channel/transporter activities, and regulation of transcription and gene expression were over-represented in the non-conserved group and are consequently under-represented in the conserved gene group.

### Comparison of Databases

Genes and the binary interactions of the proteins they encode were retrieved from three databases, IntAct, DIP, and BIND, for the five species investigated. Retired gene accession numbers were converted to updated numbers, then each accession number was converted to its Ensembl Gene ID. Only accession numbers matching the expected species were retained from each database. A summary of the data preserved from each database is presented in Additional file 2: Table S2. Unless otherwise noted, only genes annotated either "known" or "novel" by Ensembl were used. For each species, we determined the overlap between the three protein interaction databases employed (Additional file 2: Table S2). Although the number of proteins shared between any two databases is more than expected (Additional file 2: Table S2), there are few protein interactions present in all three databases, likely due to the small percentage of the proteome covered. Merging the three databases has therefore extended the PPI network coverage for each species. For example, InAct, DIP, and BIND each house protein interactions for 17, 10%, and 2.5% of the human proteome, respectively, but their combination achieved 21% coverage (28% if considering only "known" genes), highlighting the lack of knowledge in the currently available PPI networks (Additional file 2: Table S2). Yeast, however, has the largest percent of its proteome represented, with 77% present when the databases are combined, or 84% if only "known" genes are considered.

### Comparison of Interologues and Predicted Interologues

Protein interactions conserved between organisms are called interologues. Existing interologues between the five merged interactomes that we produced were identified (Additional file 3: Figure S1A). We also predicted interologues in each organism from pairs of interacting orthologues present in the other four species (Additional file 3: Figure S1B). Files have been generated for each species and are available for download as supplemental data or at http://www.interologfinder.org (listed in Additional file 4: Table S3).

We observed 99 worm interactions conserved in fly and 96 fly interactions conserved in worm, even with 25,476 known interactions in fly. Perhaps this is due to the small number of interactions known in worm, only 5204. Because of the low number of interologues between fly and worm, there were almost no interologues predicted in any species from interactions present in both of these two species. We tested the proportions of orthologues for each species of those found in the interactomes verses in the whole genomes, and found that for both fly and worm, statistically, orthologues were underrepresented in the interactomes for these two species (p-value < 0.0001). Indeed, across all species, the test for proportions of proteins with orthologues present in the interactome of each species, worm had the most extreme t-statistics of all species (t-value > 40 when compared to human, mouse, and fly versus median t-value of 22 for human, mouse, and fly when compared with each other (t-value range 19 - 55). A GO analysis was conducted on the highly conserved proteins present in the combined known and predicted interactomes (Additional file 1: Table S1). In particular, GO annotations were compared between all proteins in the known fly and worm interactomes and found to be dissimilar (data not shown). Indeed, only 20% of the "known" genes in worm have an orthologue in at least one of the other four species investigated (Figure 1). Additionally, comparing the proteins represented in the interactome and total proteome for each species revealed that proteins with orthologues are highly over-represented in known PPI.

To provide a quality measurement for protein interactions, known and predicted, we developed three confidence scores. The SpeciesScore relates both to the number of species in which an interologue has been observed and to the percent homology between orthologues known or predicted to be involved in the interaction. The ExperimentScore is based on the number of experiments in which a PPI has been observed. Finally, the InteroScore is a combination of the SpeciesScore, the ExperimentScore, and the ExperimentQualityScore. We also include the SpeciesNotation to denote the species in which an interologue is present and which species have the potential for an interologue but have not yet had one verified. The summary of this nomenclature and data available for download can be found in Additional file 4: Table S3.

Because the known interactomes for every species but yeast has a dramatic underrepresentation of the full proteome, we wanted to provide biologists with all of the predictions so that they may further investigate those potential interactions of interest to them. If a score is low, it does not mean that we have little confidence in it, only that there was only one species in which the interaction has been observed thus far. Given the poor coverage of the proteomes, this is not surprising. With this in mind, however, we wanted to assess the distribution of InteroScores in each of the five species. A cutoff of 1.5 was chosen, with those predicted interactions with an InteroScore of at least 1.5 to be those in which we have high confidence. The percentages of predicted interactions with high confidence are as follows: 50.2% for yeast, 18.4% for fly, 14.7% for worm, 11.7% for human, and 10.8% for mouse. Many of the interactions have been predicted from yeast, because it has the greatest coverage. Another factor contributing to a low score could be that the pair of predicted interacting proteins is only found in one other species, making a higher score less likely.

### Topological Analysis of PPI Networks

Many studies simply compare GO annotations between predicted interacting proteins as a benchmarking method [29]. Although GO may be used as a tool to examine a general overview of the data, we do not believe that this approach is valid as benchmarking because of the varied level and completeness of the gene annotation. Other studies compare their results to a "gold standard" [30]. Defining a gold standard for all species, considering the apparent substantial deficiency in each PPI database, however, appears inappropriate, even assuming that almost all yeast two-hybrid data is correct [31]. Given that 84% (5510/6532 known protein-coding Ensembl Gene IDs) of the yeast proteome is represented in its known interactome, we make the assumption that it is the closest to being complete. We therefore attempt to compare results from the other four species to that of yeast. Unfortunately, yeast is also the organism with the least gene conservation in the other species in this study, and this may affect the comparisons.

**Figure 2:**
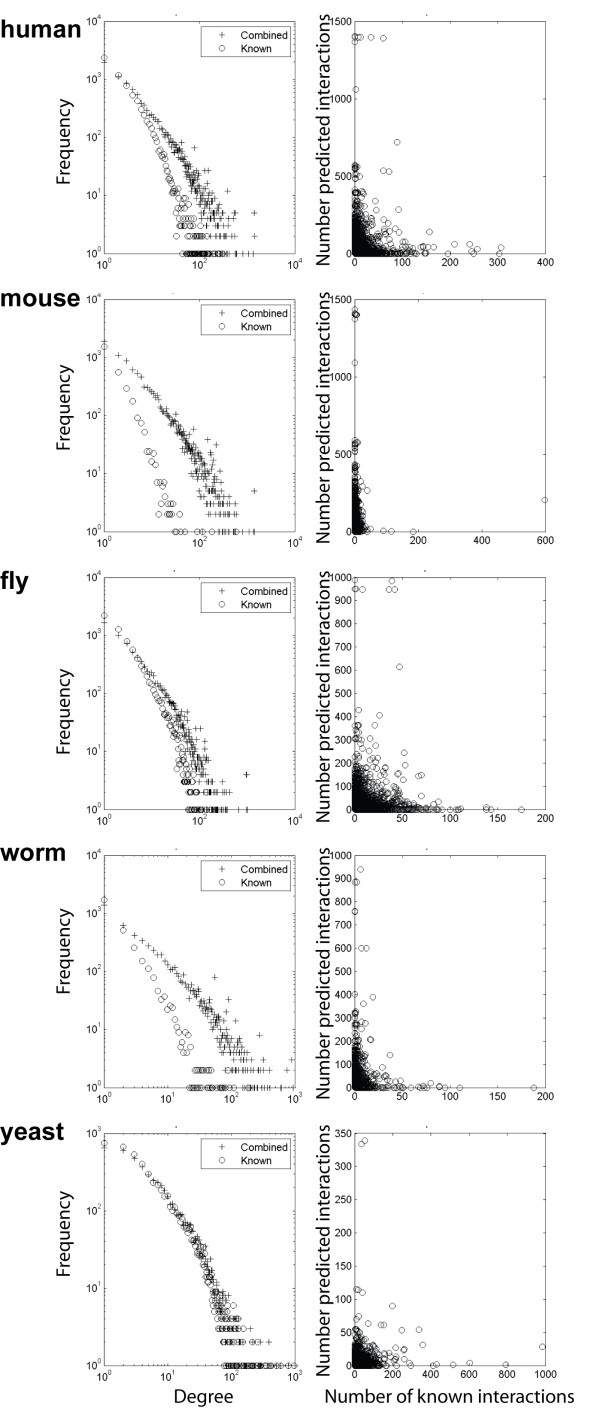
**Degree distributions **of the known (circle) and known and predicted (plus) networks (left panels) and plot of the number of known versus predicted interactions for each node (right panels) for human, mouse, fly, worm, and yeast.

To determine the effect of including predicted interactions on the PPI network structure, we analyzed their topology. We first examined the degree distribution of the networks. As can be seen in the left panels of Figure 2, like the yeast network, all networks, either with or without the predicted interactions, exhibit scale-free degree distributions (i.e. the majority of the nodes in the network have very few connections to other proteins, e.g. they have low degrees), while a small number of nodes have much higher degrees. More precisely, the degree distributions fit with the power-law function *p(k) = c k*^-*r*^. It is well known that many types of real networks, including social networks and the Internet, have scale-free degree distributions [32], which are believed to contribute to the robustness of complex systems against random node failures. The predicted interactions did not change the degree distribution for any species, which suggests that the predicted interactions are not random.

Secondly, we analyzed network diameter and clustering coefficient (Additional file 5: Figure S2A and B, respectively), which are related to the definition of small-world networks. When the known and predicted interactions are combined, for all species except yeast, the network diameters are significantly reduced and the clustering coefficients are significantly increased. With yeast, we predicted relatively few new interactions due to the robustness of the experimentally derived data, and therefore our predictions made little impact on the complete PPI network. The reduction in network diameters and increase in clustering coefficients strongly indicate that the predicted interactions increase local modularity (e.g. connecting members of a protein complex), moving closer to what is observed in the yeast known interactome. This suggestion is confirmed by our co-immunoprecipitation experiments (see below). To assess the confidence level of the results, we conducted similar tests using two random counterparts for each combined network. The first random network was obtained by randomly rewiring the combined network while preserving the degree of each node (denoted as the "rewired" network). The second random network was obtained by randomly rewiring the predicted interactions and combining them with the known interactions (denoted as the "partially rewired" network). As shown in Additional file 5: Figure S2, the partially rewired networks have diameters similar to the true combined networks, but the former have much smaller clustering coefficients. The rewired networks in all five species, on the other hand, have both smaller diameter and smaller clustering coefficients than the true combined networks. The diameter shrinkage after random rewiring often indicates a collapse of local "community" structures existing in real-world networks.

It is possible that the predicted interologues could have an over-representation of highly self-interacting protein complexes. Such a scenario would bias our network efficiency measurements. We therefore examined the efficiency of "Known" (0.0076), "Predicted" (0.0175), and "Randomized Predicted" (0.0138) human networks for each Gene Ontology (GO) slim term; the average efficiency is provided in parentheses. GO slim allows a broad view of gene ontologies for use in genome scale analysis. From these measurements, it is clear that the known human interactome is more highly connected than the predicted interactions that were added, which are no better than random interactions within the same set of proteins. Thus, the predicted interologues are represented throughout the known protein interactome and do not represent complexes or protein modules. In contrast, the connectivity (global efficiency) was improved for every slim GO term examined, with a median increase of more than 200% connectivity (data not shown). In general, increased connectivity was associated with an increased clustering coefficient (data not shown). The greatest increases in connectivity was observed in processes involved in metabolism, as might be expected considering these were the proteins most highly conserved across organisms and thus allowed the greatest number of interologue predictions. In contrast, the GO terms where we observe the least improvement in connectivity, such as extracellular space (GO:0005615), extracellular structure organization (GO:0043062) and cell surface (GO:0009986), are less well conserved across our five model organisms, and would be expected to have the least interologue predictions. In conclusion, it is expected that two proteins that are involved in the same process are likely to be more closely connected in the network, thus our predictions appear to be improving this relationship within each GO. This also suggests the predicted interologues have biological value and indicates the quality of this analysis.

In a final analysis of the networks, we compared proteins that are highly conserved across species (present in four or five of our test species) to proteins that are only moderately conserved (present in two or three of our test species) or are unique to their species. The degree distribution was measured for each group of proteins (Additional file 5: Figure S2). Weakly and highly conserved proteins have similar degree distributions in the known interactomes, but following interologue prediction, highly conserved proteins have an increased level of connectivity compared to those weakly conserved. This is expected because PPI are predicted based on interactions between conserved proteins, thus biasing the outcome. Obviously, the degree distribution of unique proteins is unchanged between known and combined interactomes, since no new interactions were predicted for these proteins.

In summary, we conclude that the combined networks have both scale-free and small-world properties, and the predicted interactions increase local modularity of the networks. Analysis of the PPI networks for all five species shows that both the known and the combined interactomes for each species exhibit scale-free degree distribution. This indicates that the predicted PPI are not random because the network structure did not change. Interestingly, we find that the number of predicted interactions for each node is inversely proportionally to the number of known interactions (Figure 2, right panels). This suggests that our method is effective in predicting PPI that have not yet been captured by experimental techniques. If a protein already has many known interactions, we may expect to predict relatively fewer interactions for it, since its interaction partners may have been extensively surveyed, either due to its popularity as a research target, or because of the non-transient nature of its interactions. But because of the extensive research previously conducted on yeast PPI, we were unable to predict many new interactions in our "gold standard" species, indicating its likely near-completeness. Again, the degree distributions for each species after prediction better resemble yeast (Additional file 5: Figure S2), suggesting that we are advancing to more complete interactomes.

### Comparison with Previous Works

There have been several studies predicting interactions; most do not include the stringent one-to-many or many-to-many orthologues as we did. Inclusion of these orthologous relationships resulting from speciation events allows for increased support of our predicted interactions. We therefore compared the predictions we made for the human interactome with those human predictions made by four earlier reports [19,22-24]. We examined the clustering coefficients and degree distributions of the four networks compared to their randomly rewired networks (range of 0.128-0.317 compared to 0.019-0.079 rewired). Similar to our predicted networks, the four other predicted networks had a higher clustering coefficient compared to that of the randomly rewired network and were similar to our benchmark of yeast. Additionally, the diameter of each was reduced when compared to the rewired network (range of 10-13 compared to 6-10 rewired), as was ours, suggesting modularity of the networks predicted by each study.

We directly compared our predicted interactions with those from STRING. To evaluate the quality of various experimental supports (see ExperimentQualityScore), we determined the ratio of interactions in both STRING and our interactome as supported by an experiment. When comparing the ratios among experiments, we discovered that the ratios among the known interactions were higher than the predicted interactions. Some ratios were dramatically different. For example in humans for MI:0397(two_hybrid_array), 185 of the 8251 (0.022) predicted interactions were included in the STRING database, however, 218 of the 237 known interactions (0.919) were included in STRING. This was not the case for all experiments. MI:0114(x-ray_crystallography) ratios of 0.427 (68 out of 159 predicted) and 0.659 (184 out of 279 known) were more similar.

### Online Tools

At http://www.interologfinder.org, we provide a link to download each set of Cytoscape files for each species (listed in Additional file 4: Table S3). In addition, we provide the ability to search for a protein or list of proteins by Ensembl Gene ID or gene name. Data retrieved from this search includes all known and predicted interactions involving the queried proteins, the InteroScore, the SpeciesNotation, and the experimental evidence for the known interactions or the evidence for the interactions from which an interologue was predicted. PPI are divided by known and predicted interactions between the queried proteins and by interactions between the queried proteins and others within the species. Data may be viewed in the user's web browser or downloaded to be directly imported into Cytoscape.

### Application 1

**Figure 3:**
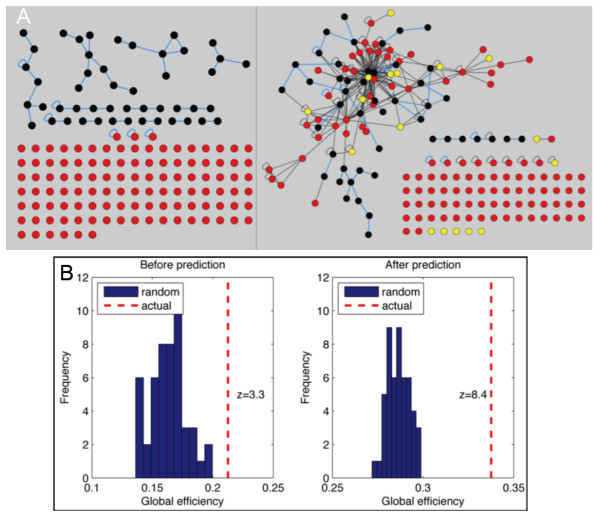
**Network analysis of proteins required for survival after MMS damage**. **A) **Subnetwork of proteins required for survival after treatment with the DNA damaging agent MMS in fly, before (left) and after (right) PPI prediction. Nodes in black are connected before prediction, while nodes in red are orphans before prediction. Yellow nodes are added to the interactome after interologue prediction. Blue edges are interactions known in fly, while black edges are predicted interactions. **B) **Global efficiency of the subnetworks before (left) and after (right) interologue prediction (dashed red line). The global efficiency of random networks determined by a set of random nodes equal to the number of nodes used in the actual set are shown with blue bars.

PPI networks can be used to reveal a higher order structure within an experimental dataset, to subsequently identify proteins and processes worth pursuing, and to derive hypotheses. We therefore employed our predicted interactome in improving the connectivity of a genomic dataset of proteins identified to be involved in damage survival [33]. We recently reported a genome-wide RNAi screen for genes required for viability after treatment with methyl methanesulfonate (MMS) in fly [34]. We validated 202 of the potential positives from the screen as being real hits; 159 are present in the known fly interactome, connected by only 42 edges (Figure 3). Seventeen proteins were present in the "predicted fly interactome" that were not present in the "known interactome," bringing the total number of proteins to 176 in the combined MMS survival subnetwork, connected by 291 edges. Forty-one of the 111 orphan proteins (nodes not connected to another node in the subnetwork) were connected to another protein after prediction. Therefore, using the predicted fly PPI network greatly increased the number of proteins in the network and the connections involved.

We used the global efficiency of the MMS survival subnetwork to examine the connectivity of the subnetwork before and after interaction prediction. The global efficiency is measured as the mean of the reciprocal of pair-wise distances and is represented by values between 0 and 1, a larger value indicating a shorter average distance, or higher connectivity. Because we expect that the predicted subnetwork will already have a much higher connectivity, as the predicted interactome did, we compared the two subnetworks with their respective randomly rewired networks. The global efficiency for the MMS subnetwork before and after prediction is 0.21 and 0.34, respectively. The Z-scores for the two measures, when compared to the same measurement in random networks, are 3.3 (p = 5e-4) and 8.4 (p < 1e-16), respectively, suggesting that the predicted PPI improved the MMS protein viability network connectivity better than by chance.

### Application 2

**Figure 4:**
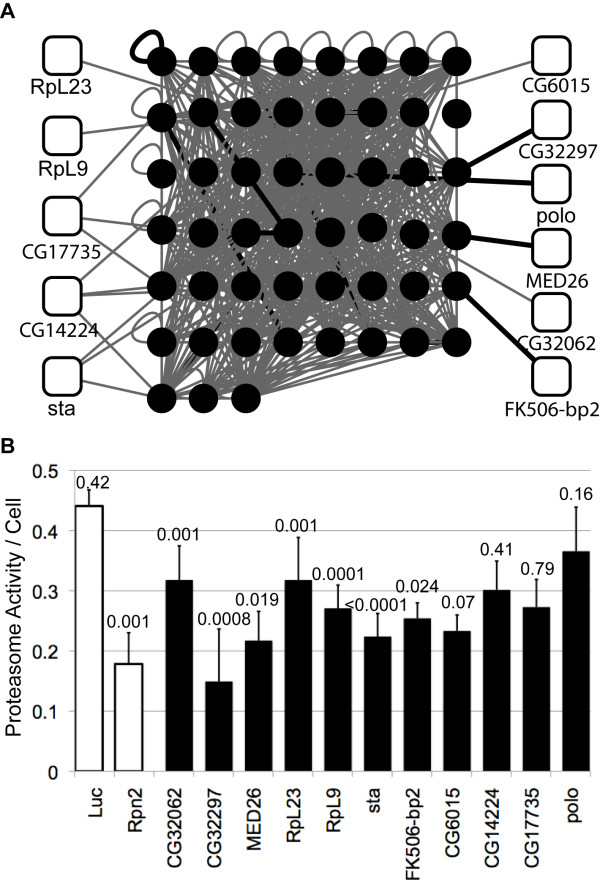
**Predicted interactors of proteasome components that affect its activity**. **A) **Subnetwork of proteasome components (black circles) and interactors (white squares) that affect proteasome activity in fly. Thick black edges are known PPI, while grey edges are predicted PPI. **B) **Proteasome activity normalized per cell after knock down of proteins indicated in **A**. Luc (non-targeting RNAi against luciferase) and Rpn2 as negative and positive controls, respectively). Error bars represent the standard deviation of four replicates, and p-values are given above each bar.

One potential advantage of using PPI networks is to facilitate the assignment of function to proteins. If the interaction of two proteins suggests that they share a role in a process, then developing improved PPI networks are of significant benefit. To test this hypothesis, we examined the role of proteins interacting with fly proteasome on proteasome activity. Forty-one members of the fly proteasome (using data from KEGG; http://www.genome.jp/) and 155 proteasome interactors are present in the "known interactome" and 409 additional interactors are predicted. Using RNA interference knock down, we individually removed each of 30 proteins that interact with proteasome components and measured proteasome activity. Knock down of seven genes resulted in a statistically significant decrease in proteasome activity (P < 0.05), while knock down of four others resulted in a decease of at least 10% in proteasome activity albeit not significantly. Of these 11 genes, four were previously known to interact with proteasome components while seven are predicted interactors (Figure 4). Among the known proteasome interactors, FK506-BP belongs to the immunophilin family involved in protein trafficking and interacts with most of the components present in the 26S proteasome [35], thus providing a "proof of principle" for this approach. Among the predicted interactors are the ribosomal proteins sta, RpL9, and RpL23. Recent investigations suggest that the proteasome might be involved in regulating translation through direct interaction with ribosomal proteins [36], which supports the interaction of these proteins, though our observation suggests a reciprocal modulation of activity. Although 19 of 30 the proteins examined did not affect proteasome function under the conditions tested, this does not imply that these proteins do not interact with proteasome components, only that their removal does not affect proteasome activity. Together these results demonstrate the utility PPI predictions and the utility of improved and expanded PPI network maps.

### Confirmation of Predicted Interactions

**Figure 5:**
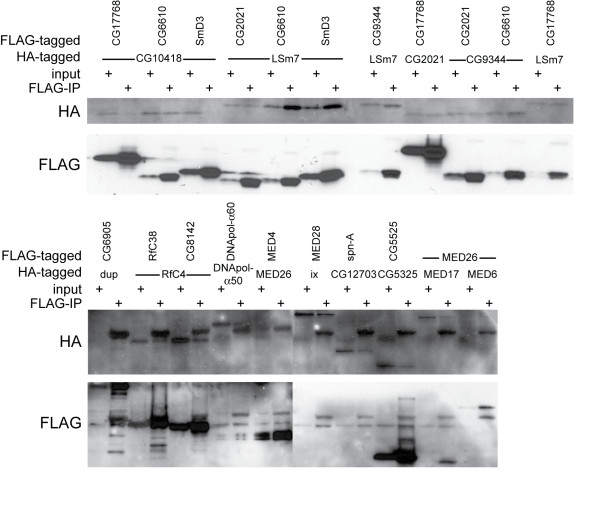
**Western blots of co-immunoprecipitations (coIP) of predicted and known interactions**. CoIP was conducted with anti-FLAG, and membranes were probed with anti-HA, stripped, and probed with anti-FLAG. Bands indicate a presence of tagged protein. "Input" is total protein before coIP, while "FLAG-IP" is protein present after coIP. If a band is present in both the input and FLAG-IP lanes, it indicates that the protein has been pulled down with the anti-FLAG antibody. When the protein in question is tagged with HA instead of FLAG, this indicates an interaction between the FLAG-tagged protein and the HA-tagged protein. InteroScores are given in parentheses: PPI between LSm7 and CG6610 (3.27), LSm7 and SmD3 (1.27), CG2021 and CG17768 (3.27), CG9344 and CG6610 (3.27), LSm7 and CG17768 (3.27), and CG12703 and spn-A (1.27) were positive controls of known PPI. Predicted PPI that were confirmed to interact were: CG10418 and CG6610 (1.39), LSm7 and CG2021 (1.69), LSm7 and CG9344 (1.39), CG9344 and CG2021 (2.69), RfC4 and RfC38 (2.64), RfC4 and CG8142 (3.27), DNApol-alpha50 and DNApol-alpha60 (1.39), MED26 and MED4 (1.18), ix and MED28 (1.18), CG5325 and CG5525 (1.39), MED17 and MED26 (1.18). Those that were not confirmed were: CG10418 and CG17768 (1.77), CG10418 and SmD3 (1.53), dup and CG6905 (1.12), and MED6 and MED26 (1.18).

In order to validate our findings of predicted interactions, we tested six known and 15 predicted fly PPI. For this we selected only proteins that have a one-to-one orthologous relationship with interacting human proteins. We selected predicted interactions in which at least one of the proteins was either known to be required for viability after MMS exposure [34] or known to interact with an MMS viability protein. Pairs of predicted interacting proteins were epitope tagged with either FLAG or HA and expressed in *Kc*_167 _cells and immunoprecipitated with FLAG-antibody. All six known protein interaction pairs were confirmed, and all but four predicted PPI tested were found to be correctly predicted (Figure 5). Some of predicted PPI are part of the mediator complex [37]. The predicted interactions in such complexes increase the local modularity of this subnetwork, and because complexes are known to exist and function as such, it is most likely that these are biologically relevant PPI. There did not seem to be a correlation between InteroScore of a predicted interaction and whether or not it was confirmed. Interestingly, three of the PPI that we predicted and validated were not predicted by STRING, namely MED26 and MED4, MED26 and MED17, and MED16 and MED2, each with an InteroScore of 1.18. All other interaction we tested, including those that we could not confirm, were predicted by STRING.

## Discussion

### PPI Prediction Across Multiple Species

Identification of PPI in multiple model organisms and in human has been accomplished by many different *in vitro *techniques, including two-hybrid expression, co-immunoprecipitation, and affinity chromatography [31,38]. Even so, a large proportion of the proteomes have not been represented in these analyses. Like several recent studies [8,19,30], we have successfully used orthologous interacting proteins from one species to predict interactions in another species. Here we have compared orthologous genes in five species (human, mouse, fly, worm, and yeast) in order to predict interologues in these organisms and created an easy to use, online tool for biologists to access the data of interest to them. Unlike comparisons conducted in other studies using only BLAST for homology, we used high quality homology annotation from Ensembl to compare orthologous genes. For orthologue determination, Ensembl has used Blastp and Smith-Waterman to compare sequences, followed by phylogenetic tree analysis, allowing one-to-many and many-to-many orthologous relationships. We found that proteins involved in metabolic processes and DNA repair were highly conserved across all species, but those proteins used in either signal transduction or cell-cell communication and transmembrane proteins were more likely to be unique to their species. Metabolism is known to be well-conserved [39], and conservation of transmembrane lies in hydrophobicity and charge probably more so than in conserved residues [40], so these discoveries are not surprising.

Unlike most PPI prediction studies predicting interaction in human only, we compared and predicted PPI in human, mouse, fly, worm, and yeast, with data retrieved from three publically available PPI databases, IntAct, DIP, and BIND. By limiting the proteins retained from these databases to those with current identifiers or convertible to current identifiers, and found only in the target species, we increase confidence in the combined PPI network. The known yeast interactome, with 5688 known or Ensembl-predicted proteins observed to be involved in 40249 interactions, is the species with the most complete protein interactome map. After prediction of interologues from the other four species, connectivity was nearly unchanged (Additional file 5 Figure S2), and as a species with nearly complete proteome coverage within its interactome (and thus our "gold standard"), we find the representation of both proteins and interactions observed in the other species sorely lacking. Our predictions for human extrapolated from other species increases the coverage from 7541 to 11647 proteins, or 32% of the proteome, or 43% of the "known" proteome. Although this is still less than half of the proteome it still includes 50% more proteins than previously included. The overlap of interologues was surprisingly little: only 1837 of the 24612 interactions observed in human had also been observed in at least one of the other species. Indeed, Gandhi *et al*. [41] only found 36 interologues overlapping between fly and worm that were not known in human, demonstrating the poor overlap in observed interactions between species. Because of the scale-free degree distribution and small-world properties of the combined interactomes, we have confidence that most of the predicted interactions are real.

### Confidence Measures

Measures akin to our SpeciesScore have been used by other researchers. Yu, *et al*. [30] reviewed two methods for determining orthologous relationships and the scoring thereof: the simplistic, albeit inaccurate, determination similarity by best-match of sequence homology versus the higher accuracy of reciprocal best-match of homology mapping. Ensembl maintains the accuracy of the reciprocal best-match, but extends it to find more complex one-to-many and many-to-many relationships based on clustering and phylogenetic trees. By using their percent identity score, we take advantage of their more complex discovery and scoring mechanism. Therefore, our SpeciesScore, exploits orthologous type descriptions given at Ensembl by scoring not just the single best orthologous pair in another species, as other researchers have done, but also other likely orthologues with slightly weaker sequence homology. We also bolster the prediction of interactions if they, or the PPI that predict them, have been found by more than one type of experiment with the ExperimentScore. When this is combined with the SpeciesScore to obtain the InteroScore, we obtain a metric that is a reasonable and intuitive solution incorporating multiple predictive PPI evidences, with higher InteroScores having more evidence supporting the interaction, either from more species or by more types of experiments. Because it appears that a large proportion of PPI are not represented in experimental data, a low score should not indicate a lack of confidence in a PPI, but rather a high score should indicate a likelihood that the interologue exists.

### Applications for Research Biologists

We have validated this methodology by verifying predicted interactions in fly by co-immunoprecipitation. Based on our interologue predictions, we have confirmed eleven new protein-protein interactions in fly. Validation of the predicted interactions was chosen not to validate our prediction method, but rather to demonstrate that predicted PPI are more likely to be real when taken together with real biological data, in this case proteins required for viability after exposure to MMS [34]. Thus, our "validation" rate is higher than expected. We therefore believe the use of interologue predictions is a powerful means to expand upon our currently available PPI network maps.

Unlike other studies, we provide a website useful to research biologists - http://www.interologfinder.org. At the website, users may not only download our data in its entirety, but also upload a list of genes of interest to them, and retrieve known and predicted interactions involving those proteins. Also included in the retrieval are InteroScores, ExperimentalEvidence, and SpeciesNotation. We have designed our output to be used with Cytoscape [28], an open source, network visualization and data integration tool that is widely adaptable.

### Suggestions for Future PPI Identification Studies

A proteome-wide analysis of interactions is unlikely to capture all possible interactions, even under the single condition in which the experiment is carried out. Braun *et al*. [38] and Venkatesan *et al*. [42] address this issue as inherent to the current high-throughput assays designed to detect PPI. Using prediction of interologues from PPI present in other species, therefore, is a promising method for research biologists to either choose pairs of proteins to test for interaction or to use as a more robust model of the interactome in order to construct hypotheses regarding their proteins of interest.

Schwartz *et al*. [43] suggest that predictions based on orthology should preferentially be used to empirically complete the interactomes cost-effectively because there is more confidence in the interactions. We believe, however, emphasis should be placed on testing PPI in which at least one protein is not conserved between species, because while these may be easily predicted, PPI between proteins with no orthology can never be predicted in this manner.

## Conclusions

Here we have predicted PPI in human and four commonly used model organisms based on orthology to proteins involved in known interactions within these five species. We provide a simple scoring mechanism, an InteroScore. This score is inclusive of the extent of homology, the number of orthologues demonstrating evidence of interaction, and the number of types of different experiments implicating the PPI. We supply our data in two formats, both readily available to the research biologist: our website http://www.interologfinder.org at which users can search for information on proteins of interest to them and also whole proteome files that may be used with the network viewer Cytoscape. Here we demonstrate two of the myriad of ways that these PPI predictions may be used to further biological research - a way to examine data from high-throughput screens (genes required for viability after MMS treatment) and to assign new function to proteins (proteins interacting with components of the proteasome). Although we are able to successfully predict PPI based on orthology, a good deal of data that cannot be predicted is still missing from these interactomes. Focus therefore must now be placed on testing interactions for those proteins.

## Methods

### Orthologue Data Collection

Orthologue data were retrieved from Ensembl 50 for all genes in *Homo sapiens*, *Mus musculus*, *Drosophila melanogaster*, *Caenorhabditis elegans*, and *Saccharomyces cerevisiae*. Ensembl has compared all genes for all species for which they have complete genome data, first against all genes of its own genome and second against all genes of every other genome in their database [25]http://www.ensembl.org/info/docs/compara/index.html. In short, Ensembl conducted the following: they used the longest translation from each gene to run both Blastp and Smith-Waterman sequence comparisons. Relationships between genes were graphed based on best reciprocal hits and best score ratios, before constructing multiple sequence alignment on each cluster that was derived. Phylogenetic trees were then constructed for each gene cluster, and these were fit with speciation trees. Finally, typical pair-wise gene relationships were determined - orthologues of one-to-one, one-to-many, and many-to-many. Additionally, Ensembl provides paralogous relationships both within and between species. These data, along with the percent sequence identities and similarities, were retrieved from Ensembl for each of the five organisms investigated from BioMart at Ensembl http://www.ensembl.org/biomart/.

A one-sample, two-tailed, hypothesis t-test for proportions was conducted for each pair-wise set of species to determine if the proportion of the interactome that had orthologues was equal to the proportion of the genome with orthologues.

### Gene Ontology Analysis

FuncAssociate [44] was used to determine the over- and under-represented GO for genes that are highly conserved or unique between the five species investigated and for proteins highly conserved that are represented in the interactomes. An adjusted p-vale of 0.05 was considered significant.

### Interactome Data Collection

Binary PPI data were retrieved from three databases: IntAct [45]http://www.ebi.ac.uk/intact/, the Database of Interacting Proteins (DIP, dip.doe-mbi.ucla.edu) [46], and the Biomolecular Interaction Network Database (BIND, bond.unleashedinformatics.com) [47]. Data was retrieved on Aug 28^th^, 2008 from all databases, and experimental evidence annotation was obtained when available.

### Conversion of Identifiers to Ensembl Gene IDs

Synonym tables provided by Ensembl (external data downloadable from their BioMart site) were used to convert the identifier for each protein used from IntAct and DIP into an Ensembl Gene ID. BIND, however, does not provide an identifier present in the Ensembl tables, but provides NCBI Gene Identifier (gi) numbers. We therefore queried NCBI [48]http://www.ncbi.nlm.nih.gov to retrieve the newest gi, and subsequently the associated NCBI protein accession numbers, for each protein. Because we were only interested in PPI, we omitted interactions involving non-protein molecules provided by BIND. For all databases, we selected only those interactions between proteins of the same species using the taxonomy identification provided. For each species, a single file with non-redundant experiments supporting the interactions was created. Gene name synonyms were also retrieved from NCBI to be used at our website, http://www.interologfinder.org, in order to accept gene names as a query.

### Interologue Prediction

For those PPI where orthologues exist for both interacting proteins in another species, but no interaction is known between those proteins in that other species, a conserved interaction (an interologue) were predicted.

### Confidence Measurements for Interologues

To achieve a single, unified confidence score for interactions and predicted interactions, we developed several scores taking into account different attributes of an interaction and combined them to form a single InteroScore.

#### OrthoScore

In order to obtain a score that represents how similar interacting partners are to their orthologues, all orthologues of a protein were combined to form a single score. Each orthologue pair (P1_s_P1_t_, where *s *is the source species and *t *is the target species) has an OrthoScore, which represents a score adjusted to represent one-to-one, one-to-many, and many-to-many relationships between P1_s _and P1_t_. For the one-to-one proteins, an OrthoScore is assigned a value of 1. This score, however, does not encompass evidence from other highly related proteins in other species, which exist as one-to-many or many-to-many orthologues, having varying degrees of identity. Orthologues which provide evidence for a predicted PPI but which have weaker homology than the highest orthologue pair are accounted for with scores having values less than 1, proportional to their percent identities as compared to the protein with the highest identity. As an example, the percent identities for three orthologues may be 92%, 87%, and 72%. The highest identity is adjusted to 100%, while the other two are scaled relative to 92% (94.5% and 78%, respectively). The values are squared to give low scores less of an effect than high scores. The resulting OrthoScore is the sum of these percents squared (1.0^2 ^+ 0.945^2 ^+ 0.78^2 ^= 2.501).

#### SingleSpeciesScore

Each annotated interologue (P1P2_s_P1P2_t_) has a Single Species Interaction Percent Identity Score (SingleSpeciesScore), formed by the product of the OrthoScores for both orthologues when the orthologues were found to interact in the target species. For example, an interaction predicted in fly is known in human with both fly proteins having a one-to-one relationship with their orthologues in human. Each OrthoScore will contribute 1.00 to the score because each is both the maximum and the only value. The combined score is 1.0 (1.00·1.00) representing the support of the interologue. However, given the more complex one-to-many example above to represent P1sP1t (2.501), combined with a one-to-one OrthoScore for P2sP2t (1.00), the resulting SingleSpeciesScore is 2.501 (2.501·1.00).

#### SpeciesScore

The SpeciesScore representing the conservation of the interaction is a combination of the number of species that support the annotated interaction, the strength of the orthologous relationships, and the number of interologues supporting the annotation. Interaction scores were not penalized for their apparent absence in another species in which the orthologues were conserved but no interaction known. In this five species analysis, a predicted interaction from only one-to-one orthologous, with the highest degree of confidence would therefore have a score of 4 (four other species have orthologues with an experimentally verified PPI). Of course if a single OrthoScore is greater than one, the overall SpeciesScore can exceed 4. For example, an interaction predicted in fly is known in human, worm, and yeast, with both fly proteins having a one-to-one relationship with their orthologues in each species. Each species will contribute 1.00 (1.00^2^) to the score because each is both the maximum and the only value. The final SpeciesScore would be 3.00 (1.00 + 1.00 + 1.00) and reflects the number of species supporting the interaction. A more complex example is an interaction predicted in fly that is known in worm as a single interaction (again, with all proteins being one-to-one orthologues), but is present in human as three interologues due to paralogues in human (in a one-to-many or many-to-many orthologous relationship with fly proteins). The fly interaction would therefore score 1.00 from worm, but higher from human. Using the one-to-many example above, the final SpeciesScore for this second example fly predicted interaction is therefore 3.501 (1.00 + 2.501). Contrasting these two examples of high support for the predictions, one is due to the diverse number of species that support the prediction and the other due to the number of interologues within a single species that support the prediction.

#### ExperimentScore

Using the experimental evidence available from DIP and IntAct (experiment identifier and type), the ExperimentScore was developed. For each PPI, each type of experimental evidence was only counted once regardless of the number of databases in which it was found, and the total number of experiments for each PPI is the ExperimentScore. BIND does not include experiment type in its publicly available database and was therefore generally excluded from this confidence score; PPI annotated only in BIND were described as "protein_protein" and given an ExperimentScore of 1. Cytoscape files were created for the annotated interactions. The following equation was used, where N is the count of all experiments that support the interaction between P1 and P2.

#### ExperimentQualityScore

To quantify the quality of various experimental evidences, we determined how many interactions annotated or predicted by a specific experiment were also present in STRING. For a given experiment, such as MI:0019(coimmunoprecipitation) in worm, we determined that out of 4324 interactions annotated by this experiment only 813 of these interactions were present in the STRING database for a ratio of 0.188 (813/4324). Conversely, 4287 interactions out of 5971 MI:0398(two_hybrid_pooling) annotated interactions were found in STRING for a ratio of 0.718. To incorporate as much evidence as possible while adjusting for quality of experimentation support, the ratios for a single interaction's experimental evidence were summed. Thus, an interaction described by both MI:0019 and MI:0398 would have an ExperimentQualityScore of 0.906 (0.188+0.718).

#### InteroScore

To create a single, combined confidence measurement, we took the product of the SpeciesScore and the ExperimentScore then added the ExperimentQualityScore for each interaction and refer to it as the InteroScore.

#### SpeciesNotation

To provide users with a quick indication of the species in which each PPI is found and the species in which each has the potential to exist, we developed the SpeciesNotation. We used the first letter of the genus, with uppercase indicating that the PPI exists between orthologues in that species, and lowercase indicating the existence of orthologues in that species but no confirmed interaction.

### Data Output

The data is presented in a format compatible with the interaction viewer Cytoscape [28]http://cytoscape.org/. For each species, these files include a PPI file, files noting the InteroScore, SpeciesScore, ExperimentScore, SpeciesNotation, and the type of orthology (Additional file 4: Table S3, Additional file 6: Zip Hsap, Additional file 7: Zip Mmus, Additional file 8: Zip Dmel, Additional file 9: Zip Cele, Additional file 10: Zip Scer).

### Connectivity Measurements

Briefly, the shortest path between two given nodes in a network is the minimum number of edges that need to be traversed to get from one node to the other; the diameter of a network is the maximum length among all shortest paths between any two nodes in the network. Many real-world networks as well as random networks (e.g., the canonical Erdos-Ryni network [32]) have small diameters, while highly structured networks, such as high-dimensional grids typically have large diameters [32]. On the other hand, real-world networks often differ from random networks in their clustering coefficient, which is defined as *c = 3(tri/conn_trip)*, where tri is the number of triangles in the graph and conn_trip is a "connected triple" or a path of three nodes. For many real-world networks, the clustering coefficient is between 0.1 and 0.5, while for random networks (such as the Erdos-Ryni network) of n nodes, lim_n→∞ _c = 0. A high clustering coefficient indicates that the network is organized into modules, and networks with both high clustering coefficients and small diameters are collectively called small-world networks [32].

### Media, Strains, and Plasmid Constructs

*Drosophila Kc*_167 _cells were maintained in Schneider's medium as previously described [33]. Genes were cloned from fly genomic DNA purified from *Kc*_167 _cells (Table 1). If the first round of cloning failed, genes were cloned from cDNA reverse transcribed using the ImProm-II Reverse Transcription System (Promega, Madison, WI) from RNA obtained from whole flies (generously provided by Dr. Donald McEwen, UTHSCSA). PCR was conducted using gene specific primers designed to the 5' and 3' ends of the transcripts to be cloned. The 5' end of each forward primer was designed as CACCATG in order to directionally clone it into GATEWAY destination vectors, with the ATG in frame for transcription. The reactions were 0.2 μM each primer, 0.2 mM dNTPs, 2 mM MgSO_4_, 1× high fidelity buffer, 1 U Platinum Taq (Invitrogen), with 35 cycles per manufacturer's instructions. PCR products were TOPO TA cloned into pENTR/D-TOPO (Invitrogen) per manufacturer's instructions. Resultant plasmids were bidirectionally sequenced with M13 primers. Clones were then moved to one or more of the following GATEWAY vectors, pAFW and pAWF (3xFLAG) or pAHW and pAWH (3xHA) [49] per manufacturer's instructions. A GFP-expressing plasmid, pAB27, was constructed as a control for transfection; EGFP-N1 (Clontech, BD Biosciences, San Jose, CA) was digested with *Kpn*I and *Not*I, and the fragment containing EGFP was directionally cloned into pAct5 (Invitrogen), which had also been digested with *Kpn*I and *Not*I.

### Transfection and Expression

For single transfections, *Kc*_167 _cells were plated at 2 × 10^6 ^cells at 1 mL/well in 6-well plates. Cellfectin (Invitrogen) was added at 7 μL/mL cultured *Kc*_167 _cells with 1 μg DNA per manufacturer's protocol. pAB27 was used as a transfection control. All plasmids were cotransfected with 0.1 μg pCoHygro (Invitrogen) for hygromycin resistance; hygromycin (Roche, Indianapolis, IN) was added 48 hours after transfection at 300 μg/mL. After an additional three days, medium was changed and 150 μg/mL hygromycin was added. For cotransfections, 0.5 μg of each plasmid was transfected along with pCoHygro. Cells were plated in 100 mm dishes in 2 mL of medium.

### Protein Purification, Immunoprecipitation, Separation, and Detection

Cells were harvested in cold PBS, and protein lysates were prepared using radioimmunoprecipitation assay buffer (50 mM Tris pH 7.4, 150 mM NaCl, 0.1% w/v SDS, 0.5% w/v sodium deoxycholate, 1% w/v NP40, and the protease inhibitors (Sigma) 0.2 U/mL Aprotinin, 1 mM phenylmethanesufonyl fluoride, and 1 mM Na_3_VO_4_), and protein concentration was determined using a Bio-Rad Protein Assay Kit (Bio-Rad, Hercules, CA). Protein was immunoprecipitated with anti-FLAG (Sigma) as described at http://cellsignal.com/. Equal amounts of proteins were resolved by 8% or 12% SDS-polyacrylamide gel electrophoresis. Proteins were transferred to membrane and detected using the ONE-HOUR Western Complete Kit (GenScript, Piscataway, NJ), including their secondary antibody and detection per manufacturer's protocol. Either mouse anti-HA (Convance, Quebec) or rabbit anti-FLAG (Sigma) at 1:100 were used as the primary antibody. Two additional washes with 1× TBST, pH 7.6 were employed before the wash dictated by the protocol.

### Proteasome Assay

Proteasome activity was measured in *Drosophila Kc*_167 _cells with a cell based Proteasome-Glo assay kit (Promega), using methods described before [34]. dsRNA were produced also as previously described [33].

## Authors' contributions

AMW obtained data from the outside sources, conducted the comparisons between species, networks and annotations, designed the data and website output, analyzed the results from the "applications," coordinated all work on the project, and drafted the paper. MD reconciled accession numbers to current updates and Ensembl Gene IDs, integrated data from the interactome databases, and computed predicted PPI and scores and assisted in writing. JR conducted the network analysis, including clustering coefficients and degree analysis and assisted in writing. TTG carried out the gene cloning and co-IP experiments, and DR carried out the proteasome experiments. BB organized the data for output files and wrote the code for the website. AJRB designed the project and assisted in writing the manuscript. All authors read and approved the final manuscript.

## Supplementary Material

Additional file 1 **Supplemental Table 1. GO Analyses**. A Gene Ontology analysis of genes unique to their species and genes that are conserved in all five species in this study, and a Gene Ontology analysis of highly conserved proteins that are represented in interactomes of the five species in this study. Click here for file

Additional file 2 **Supplemental Table 2. Descriptive Statistics of Interaction Databases**. The number of genes and interactions unique to each databases and total genes and interactions represented in each databases is shown. The number of genes and interactions lost from each database after conversion to unique Ensembl Gene IDs is also indicated. For human data, the gene and interaction overlap (after conversion to Ensembl Gene ID) is given as observed and expected values. Click here for file

Additional file 3 **Supplemental Figure 1. Four-way Venn Diagrams of interologues in five species**. For each species, human (red), mouse (orange), fly (yellow), worm (green), and yeast (blue), **A) **interologues known to be conserved by experimental evidence in the other four species are shown. For example, there are 22775 interactions in human that are not known to be conserved in any of the other four species, but there are 573 human interactions that are conserved in yeast, and 50 human interactions conserved in yeast and mouse together. **B) **Interologues predicted in each organism by orthology noted by the species from which the predictions are based. Click here for file

Additional file 4 **Supplemental Table 3**. File Types Available for Download in Supplementary Material. Click here for file

Additional file 5 **Supplemental Figure 2**. **A) **Diameters and **B) **clustering coefficients for worm (ce), fly (dm), human (hs), mouse (mm), and yeast (sc). Metrics for the combined known and predicted interactions (dark blue), known (cyan), a random network obtained by randomly rewiring the predicted interactions and combining them with the known interactions (partially rewired, yellow), and a random network obtained by randomly rewiring the combined network while preserving the degree of each node (rewired, red) are shown. Click here for file

Additional file 6 **Zip Hsap**. A zipped folder containing files formatted for use in Cytoscape listed in Supplemental Table 3. These files are for *Homo sapiens *proteins. Click here for file

Additional file 7 **Zip Mmus**. A zipped folder containing files formatted for use in Cytoscape listed in Supplemental Table 3. These files are for *Mus musculus *proteins. Click here for file

Additional file 8 **Zio Dmel**. A zipped folder containing files formatted for use in Cytoscape listed in Supplemental Table 3. These files are for *Drosophila melanogaster *proteins. Click here for file

Additional file 9 **Zip Cele**. A zipped folder containing files formatted for use in Cytoscape listed in Supplemental Table 3. These files are for *Caenorhabditis elegans *proteins. Click here for file

Additional file 10 **Zip Cele**. A zipped folder containing files formatted for use in Cytoscape listed in Supplemental Table 3. These files are for *Caenorhabditis elegans *proteins. Click here for file

## References

[B1] Wang L Tu Z Sun F A network-based integrative approach to prioritize reliable hits from multiple genome-wide RNAi screens in Drosophila BMC Genomics 2009 10 220 10.1186/1471-2164-10-220 19435510 PMC2697172

[B2] Fromont-Racine M Rain JC Legrain P Toward a functional analysis of the yeast genome through exhaustive two-hybrid screens Nat Genet 1997 16 277 282 10.1038/ng0797-277 9207794

[B3] Uetz P Giot L Cagney G Mansfield TA Judson RS Knight JR Lockshon D Narayan V Srinivasan M Pochart P A comprehensive analysis of protein-protein interactions in Saccharomyces cerevisiae Nature 2000 403 623 627 10.1038/35001009 10688190

[B4] Walhout AJ Sordella R Lu X Hartley JL Temple GF Brasch MA Thierry-Mieg N Vidal M Protein interaction mapping in C. elegans using proteins involved in vulval development Science 2000 287 116 122 10.1126/science.287.5450.116 10615043

[B5] Ito T Chiba T Ozawa R Yoshida M Hattori M Sakaki Y A comprehensive two-hybrid analysis to explore the yeast protein interactome Proc Natl Acad Sci USA 2001 98 4569 4574 10.1073/pnas.061034498 11283351 PMC31875

[B6] Giot L Bader JS Brouwer C Chaudhuri A Kuang B Li Y Hao YL Ooi CE Godwin B Vitols E A protein interaction map of Drosophila melanogaster Science 2003 302 1727 1736 10.1126/science.1090289 14605208

[B7] Reboul J Vaglio P Rual JF Lamesch P Martinez M Armstrong CM Li S Jacotot L Bertin N Janky R C. elegans ORFeome version 1.1: experimental verification of the genome annotation and resource for proteome-scale protein expression Nat Genet 2003 34 35 41 10.1038/ng1140 12679813

[B8] Li S Armstrong CM Bertin N Ge H Milstein S Boxem M Vidalain PO Han JD Chesneau A Hao T A map of the interactome network of the metazoan C. elegans Science 2004 303 540 543 10.1126/science.1091403 14704431 PMC1698949

[B9] Colland F Jacq X Trouplin V Mougin C Groizeleau C Hamburger A Meil A Wojcik J Legrain P Gauthier JM Functional proteomics mapping of a human signaling pathway Genome Res 2004 14 1324 1332 10.1101/gr.2334104 15231748 PMC442148

[B10] Rual JF Venkatesan K Hao T Hirozane-Kishikawa T Dricot A Li N Berriz GF Gibbons FD Dreze M Ayivi-Guedehoussou N Towards a proteome-scale map of the human protein-protein interaction network Nature 2005 437 1173 1178 10.1038/nature04209 16189514

[B11] Stelzl U Worm U Lalowski M Haenig C Brembeck FH Goehler H Stroedicke M Zenkner M Schoenherr A Koeppen S A human protein-protein interaction network: a resource for annotating the proteome Cell 2005 122 957 968 10.1016/j.cell.2005.08.029 16169070

[B12] Yu H Braun P Yildirim MA Lemmens I Venkatesan K Sahalie J Hirozane-Kishikawa T Gebreab F Li N Simonis N High-quality binary protein interaction map of the yeast interactome network Science 2008 322 104 110 10.1126/science.1158684 18719252 PMC2746753

[B13] Altschul SF Madden TL Schaffer AA Zhang J Zhang Z Miller W Lipman DJ Gapped BLAST and PSI-BLAST: a new generation of protein database search programs Nucleic Acids Res 1997 25 3389 3402 10.1093/nar/25.17.3389 9254694 PMC146917

[B14] Smith TF Waterman MS Identification of common molecular subsequences J Mol Biol 1981 147 195 197 10.1016/0022-2836(81)90087-5 7265238

[B15] Goffard N Garcia V Iragne F Groppi A de Daruvar A IPPRED: server for proteins interactions inference Bioinformatics 2003 19 903 904 10.1093/bioinformatics/btg091 12724307

[B16] Han K Park B Kim H Hong J Park J HPID: the Human Protein Interaction Database Bioinformatics 2004 20 2466 2470 10.1093/bioinformatics/bth253 15117749

[B17] Huang TW Tien AC Huang WS Lee YC Peng CL Tseng HH Kao CY Huang CY POINT: a database for the prediction of protein-protein interactions based on the orthologous interactome Bioinformatics 2004 20 3273 3276 10.1093/bioinformatics/bth366 15217821

[B18] Lehner B Fraser AG A first-draft human protein-interaction map Genome Biol 2004 5 R63 10.1186/gb-2004-5-9-r63 15345047 PMC522870

[B19] Brown KR Jurisica I Online predicted human interaction database Bioinformatics 2005 21 2076 2082 10.1093/bioinformatics/bti273 15657099

[B20] Persico M Ceol A Gavrila C Hoffmann R Florio A Cesareni G HomoMINT: an inferred human network based on orthology mapping of protein interactions discovered in model organisms BMC Bioinformatics 2005 6 Suppl 4 S21 10.1186/1471-2105-6-S4-S21 16351748 PMC1866386

[B21] Rhodes DR Tomlins SA Varambally S Mahavisno V Barrette T Kalyana-Sundaram S Ghosh D Pandey A Chinnaiyan AM Probabilistic model of the human protein-protein interaction network Nat Biotechnol 2005 23 951 959 10.1038/nbt1103 16082366

[B22] Michaut M Kerrien S Montecchi-Palazzi L Chauvat F Cassier-Chauvat C Aude JC Legrain P Hermjakob H InteroPORC: automated inference of highly conserved protein interaction networks Bioinformatics 2008 24 1625 1631 10.1093/bioinformatics/btn249 18508856

[B23] Jensen LJ Kuhn M Stark M Chaffron S Creevey C Muller J Doerks T Julien P Roth A Simonovic M STRING 8--a global view on proteins and their functional interactions in 630 organisms Nucleic Acids Res 2009 37 D412 416 10.1093/nar/gkn760 18940858 PMC2686466

[B24] Huang TW Lin CY Kao CY Reconstruction of human protein interolog network using evolutionary conserved network BMC Bioinformatics 2007 8 152 10.1186/1471-2105-8-152 17493278 PMC1885812

[B25] Vilella AJ Severin J Ureta-Vidal A Heng L Durbin R Birney E EnsemblCompara GeneTrees: Complete, duplication-aware phylogenetic trees in vertebrates Genome Res 2009 19 327 335 10.1101/gr.073585.107 19029536 PMC2652215

[B26] Oti M Snel B Huynen MA Brunner HG Predicting disease genes using protein-protein interactions J Med Genet 2006 43 691 698 10.1136/jmg.2006.041376 16611749 PMC2564594

[B27] Goh KI Cusick ME Valle D Childs B Vidal M Barabasi AL The human disease network Proc Natl Acad Sci USA 2007 104 8685 8690 10.1073/pnas.0701361104 17502601 PMC1885563

[B28] Shannon P Markiel A Ozier O Baliga NS Wang JT Ramage D Amin N Schwikowski B Ideker T Cytoscape: a software environment for integrated models of biomolecular interaction networks Genome Res 2003 13 2498 2504 10.1101/gr.1239303 14597658 PMC403769

[B29] Espadaler J Romero-Isart O Jackson RM Oliva B Prediction of protein-protein interactions using distant conservation of sequence patterns and structure relationships Bioinformatics 2005 21 3360 3368 10.1093/bioinformatics/bti522 15961445

[B30] Yu H Luscombe NM Lu HX Zhu X Xia Y Han JD Bertin N Chung S Vidal M Gerstein M Annotation transfer between genomes: protein-protein interologs and protein-DNA regulogs Genome Res 2004 14 1107 1118 10.1101/gr.1774904 15173116 PMC419789

[B31] Cusick ME Yu H Smolyar A Venkatesan K Carvunis AR Simonis N Rual JF Borick H Braun P Dreze M Literature-curated protein interaction datasets Nat Methods 2009 6 39 46 10.1038/nmeth.1284 19116613 PMC2683745

[B32] Newman MEJ The structure and function of complex networks SIAM Review 2003 45 167 256 10.1137/S003614450342480

[B33] Wiles AM Ravi D Bhavani S Bishop AJ An analysis of normalization methods for Drosophila RNAi genomic screens and development of a robust validation scheme J Biomol Screen 2008 13 777 784 10.1177/1087057108323125 18753689 PMC2956424

[B34] Ravi D Wiles AM Bhavani S Ruan J Leder P Bishop AJ A network of conserved damage survival pathways revealed by a genomic RNAi screen PLoS Genet 2009 5 e1000527 10.1371/journal.pgen.1000527 19543366 PMC2688755

[B35] Nakagawa T Shirane M Iemura S Natsume T Nakayama KI Anchoring of the 26S proteasome to the organellar membrane by FKBP38 Genes Cells 2007 12 709 719 17573772 10.1111/j.1365-2443.2007.01086.x

[B36] Caldarola S De Stefano MC Amaldi F Loreni F Synthesis and function of ribosomal proteins--fading models and new perspectives FEBS J 2009 276 3199 3210 10.1111/j.1742-4658.2009.07036.x 19438715

[B37] Kim YJ Lis JT Interactions between subunits of Drosophila Mediator and activator proteins Trends Biochem Sci 2005 30 245 249 10.1016/j.tibs.2005.03.010 15896742

[B38] Braun P Tasan M Dreze M Barrios-Rodiles M Lemmens I Yu H Sahalie JM Murray RR Roncari L de Smet AS An experimentally derived confidence score for binary protein-protein interactions Nat Methods 2009 6 91 97 10.1038/nmeth.1281 19060903 PMC2976677

[B39] Smith E Morowitz HJ Universality in intermediary metabolism Proc Natl Acad Sci USA 2004 101 13168 13173 10.1073/pnas.0404922101 15340153 PMC516543

[B40] Elofsson A von Heijne G Membrane protein structure: prediction versus reality Annu Rev Biochem 2007 76 125 140 10.1146/annurev.biochem.76.052705.163539 17579561

[B41] Gandhi TK Zhong J Mathivanan S Karthick L Chandrika KN Mohan SS Sharma S Pinkert S Nagaraju S Periaswamy B Analysis of the human protein interactome and comparison with yeast, worm and fly interaction datasets Nat Genet 2006 38 285 293 10.1038/ng1747 16501559

[B42] Venkatesan K Rual JF Vazquez A Stelzl U Lemmens I Hirozane-Kishikawa T Hao T Zenkner M Xin X Goh KI An empirical framework for binary interactome mapping Nat Methods 2009 6 83 90 10.1038/nmeth.1280 19060904 PMC2872561

[B43] Schwartz AS Yu J Gardenour KR Finley RL Jr Ideker T Cost-effective strategies for completing the interactome Nat Methods 2009 6 55 61 10.1038/nmeth.1283 19079254 PMC2613168

[B44] Berriz GF King OD Bryant B Sander C Roth FP Characterizing gene sets with FuncAssociate Bioinformatics 2003 19 2502 2504 10.1093/bioinformatics/btg363 14668247

[B45] Hermjakob H Montecchi-Palazzi L Lewington C Mudali S Kerrien S Orchard S Vingron M Roechert B Roepstorff P Valencia A IntAct: an open source molecular interaction database Nucleic Acids Res 2004 32 D452 455 10.1093/nar/gkh052 14681455 PMC308786

[B46] Xenarios I Salwinski L Duan XJ Higney P Kim SM Eisenberg D DIP, the Database of Interacting Proteins: a research tool for studying cellular networks of protein interactions Nucleic Acids Res 2002 30 303 305 10.1093/nar/30.1.303 11752321 PMC99070

[B47] Alfarano C Andrade CE Anthony K Bahroos N Bajec M Bantoft K Betel D Bobechko B Boutilier K Burgess E The Biomolecular Interaction Network Database and related tools 2005 update Nucleic Acids Res 2005 33 D418 424 10.1093/nar/gki051 15608229 PMC540005

[B48] Maglott D Ostell J Pruitt KD Tatusova T Entrez Gene: gene-centered information at NCBI Nucleic Acids Res 2007 35 D26 31 10.1093/nar/gkl993 17148475 PMC1761442

[B49] The Drosophila Gateway Vector Collection http://www.ciwemb.edu/labs/murphy/Gateway%20vectors.html

