# Supplementary figures and images for: Building and analyzing protein interactome networks by cross-species comparisons
Source: BMC Syst Biol. 2010 Mar 30;4:36. doi: 10.1186/1752-0509-4-36 (PMC2859380; doi:10.1186/1752-0509-4-36)

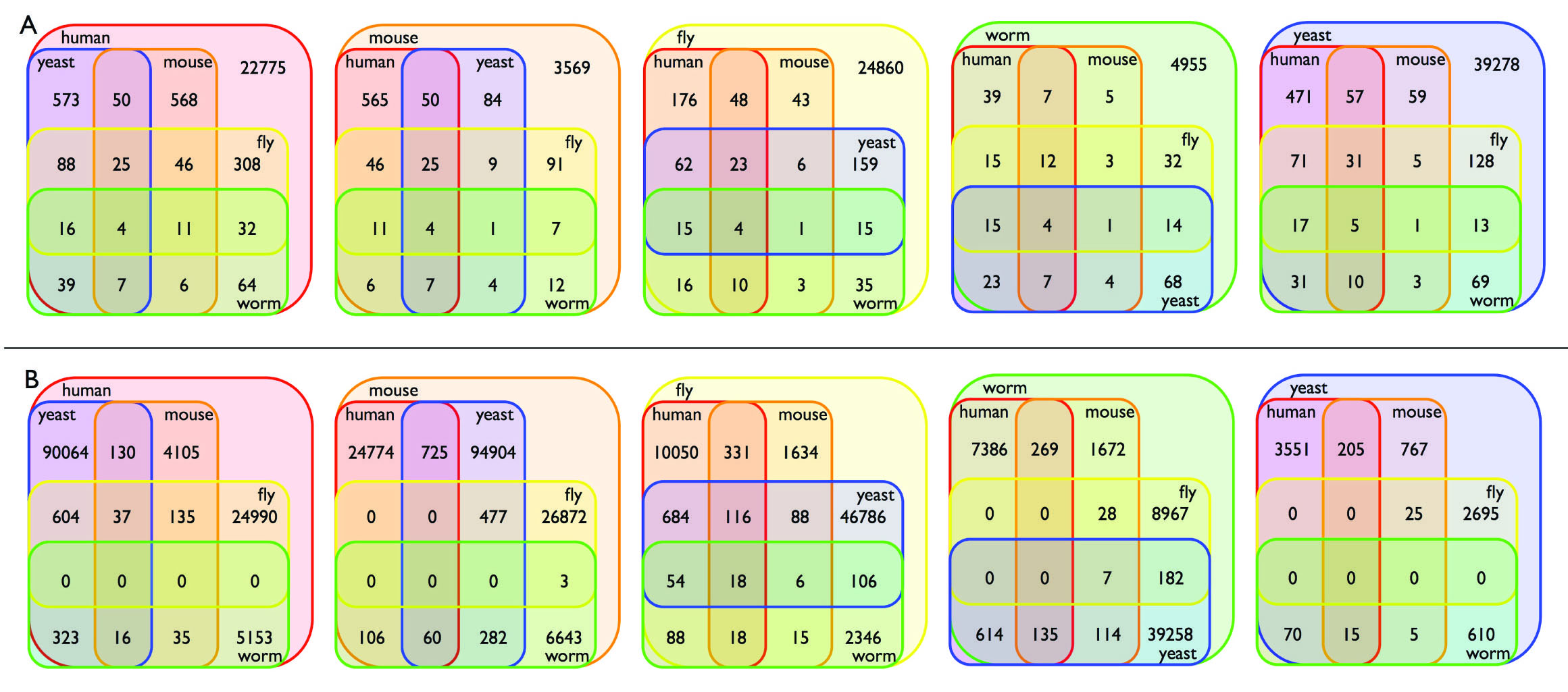

Supplement: Additional file 3 — Supplemental Figure 1. Four-way Venn Diagrams of interologues in five species. For each species, human (red), mouse (orange), fly (yellow), worm (green), and yeast (blue), A) interologues known to be conserved by experimental evidence in the other four species are shown. For example, there are 22775 interactions in human that are not known to be conserved in any of the other four species, but there are 573 human interactions that are conserved in yeast, and 50 human interactions conserved in yeast and mouse together. B) Interologues predicted in each organism by orthology noted by the species from which the predictions are based. [file 1752-0509-4-36-S3.JPEG]

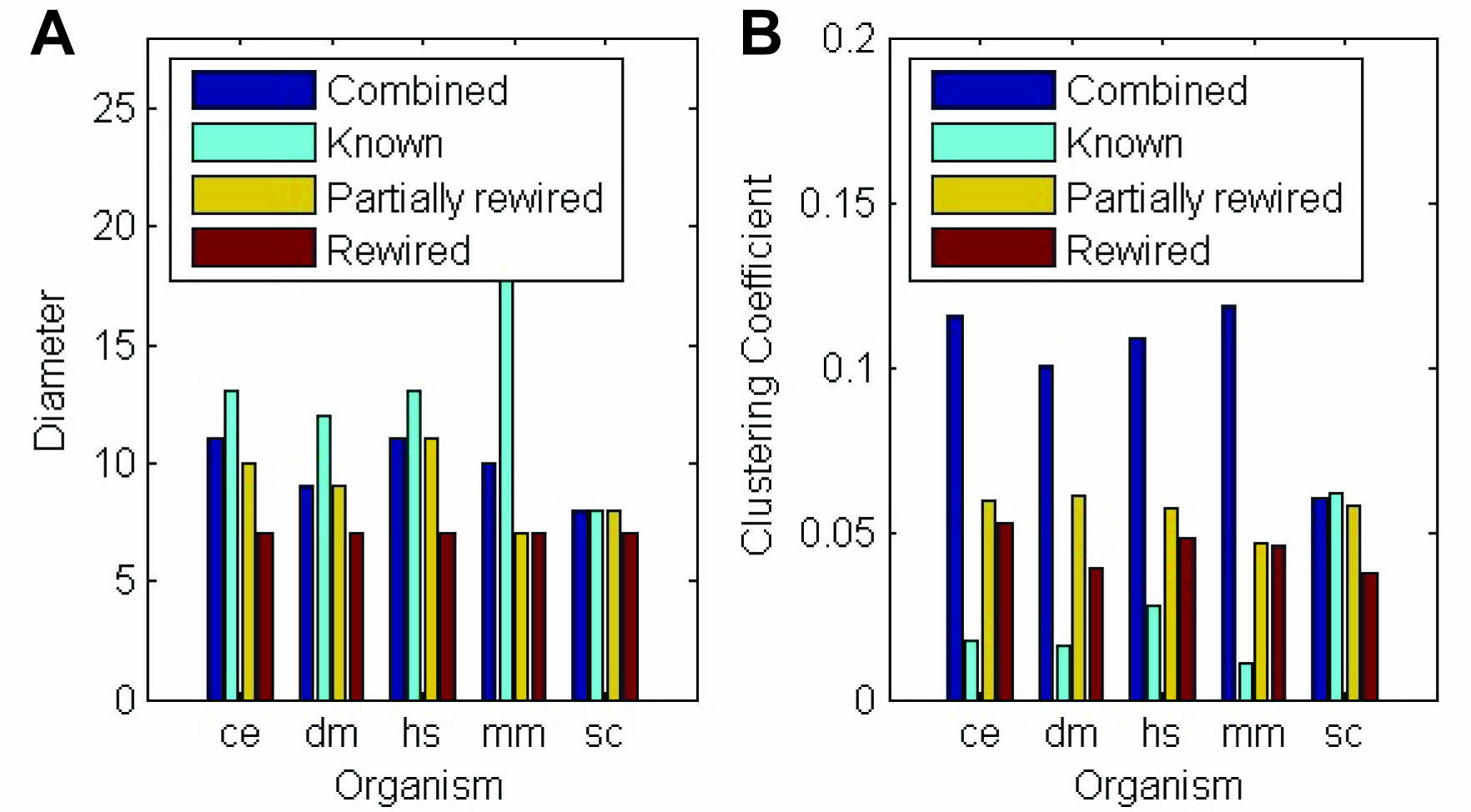

Supplement: Additional file 5 — Supplemental Figure 2. A) Diameters and B) clustering coefficients for worm (ce), fly (dm), human (hs), mouse (mm), and yeast (sc). Metrics for the combined known and predicted interactions (dark blue), known (cyan), a random network obtained by randomly rewiring the predicted interactions and combining them with the known interactions (partially rewired, yellow), and a random network obtained by randomly rewiring the combined network while preserving the degree of each node (rewired, red) are shown. [file 1752-0509-4-36-S5.JPEG]
